# Supplementary material for: Smooth Interpolating Curves with Local Control and Monotone Alternating Curvature
Source: Comput Graph Forum. 2022 Oct 6;41(5):25–38. doi: 10.1111/cgf.14600 (PMC9827861; doi:10.1111/cgf.14600)
Supplement: Supplementary file 1 — Supplement Material [file CGF-41-25-s001.zip › Local-Smooth-Interpolating-MonoCurvature/extern/clothoids/submodules/Utils/docs/api-cpp/function_a00082_1a934692c46987c2baa575b77885abfbec.html]

Function Utils::has\_SSE42 — Utils v1.2

### Navigation

- index
- toc
- next
- previous
- Utils »
- C++ API »
- Function Utils::has\_SSE42

# Function Utils::has\_SSE42¶

- Defined in File Utils\_CPU\_info.hh

## Function Documentation¶

bool Utils::has\_SSE42()¶
:   check if CPU support SSE42 instruction set

### Quick search

### Table of Contents

- rang - Colors for your Terminal
- Zstr
- C++ API

«
hide menu

menu
sidebar
»

### Navigation

- index
- toc
- next
- previous
- Utils »
- C++ API »
- Function Utils::has\_SSE42

© Copyright 2021, Enrico Bertolazzi.
Created using Sphinx 4.2.0.
